# Supplementary figures and images for: Pre-clinical studies of Schistosoma mansoni vaccines: A scoping review
Source: PLoS Negl Trop Dis. 2025 Jun 2;19(6):e0012956. doi: 10.1371/journal.pntd.0012956 (PMC12158002; doi:10.1371/journal.pntd.0012956)

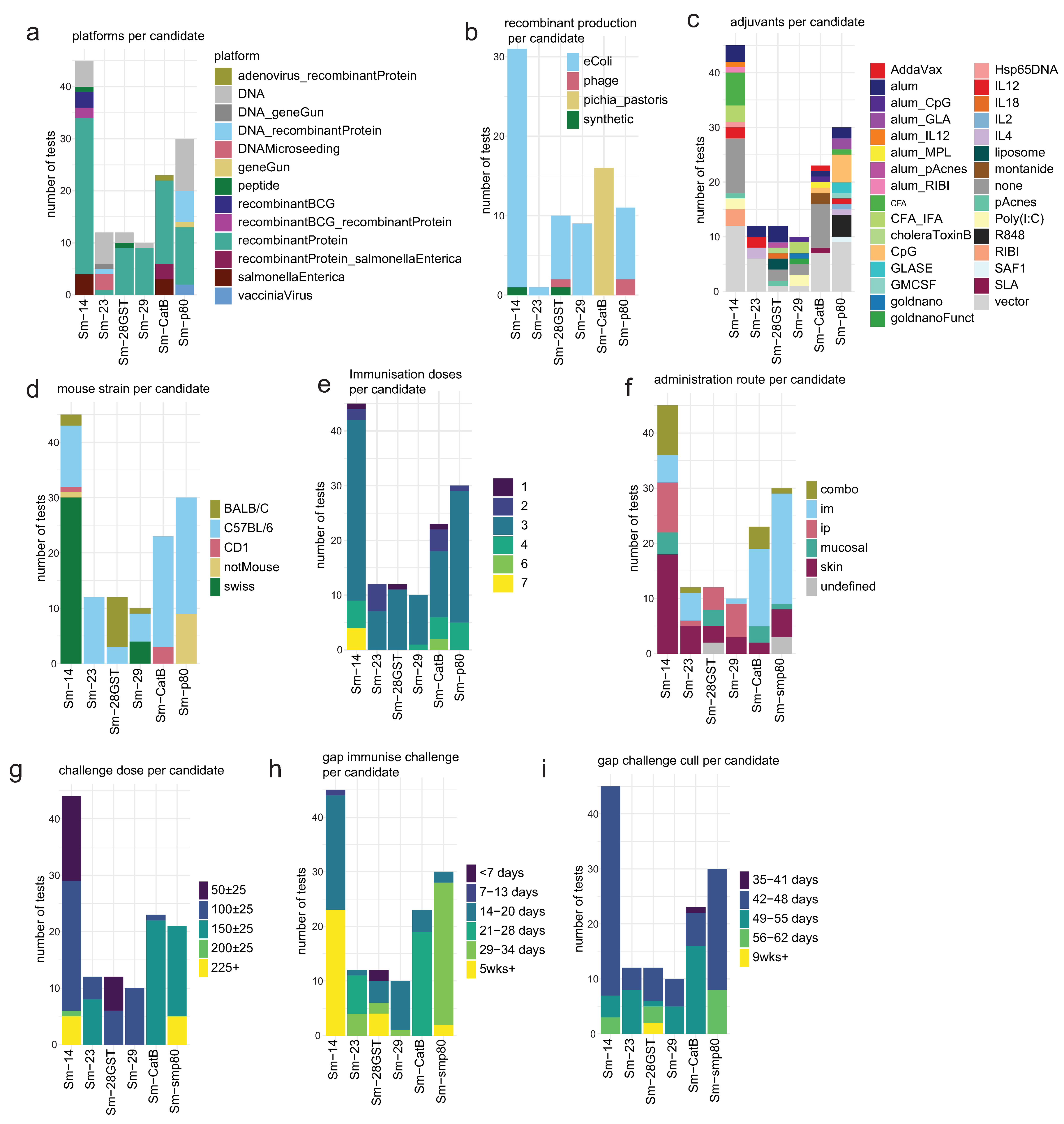

Supplement: S1 Fig — Stacked barplots show the number of tests with the indicated parameter a) platforms, b) recombinant protein production, c) adjuvants, d) mouse strain, e) immunisation doses, f) administration route, g) challenge dose, h) gap immunisation and challenge, i) gap challenge and cull. (PDF) [file pntd.0012956.s002.tif]

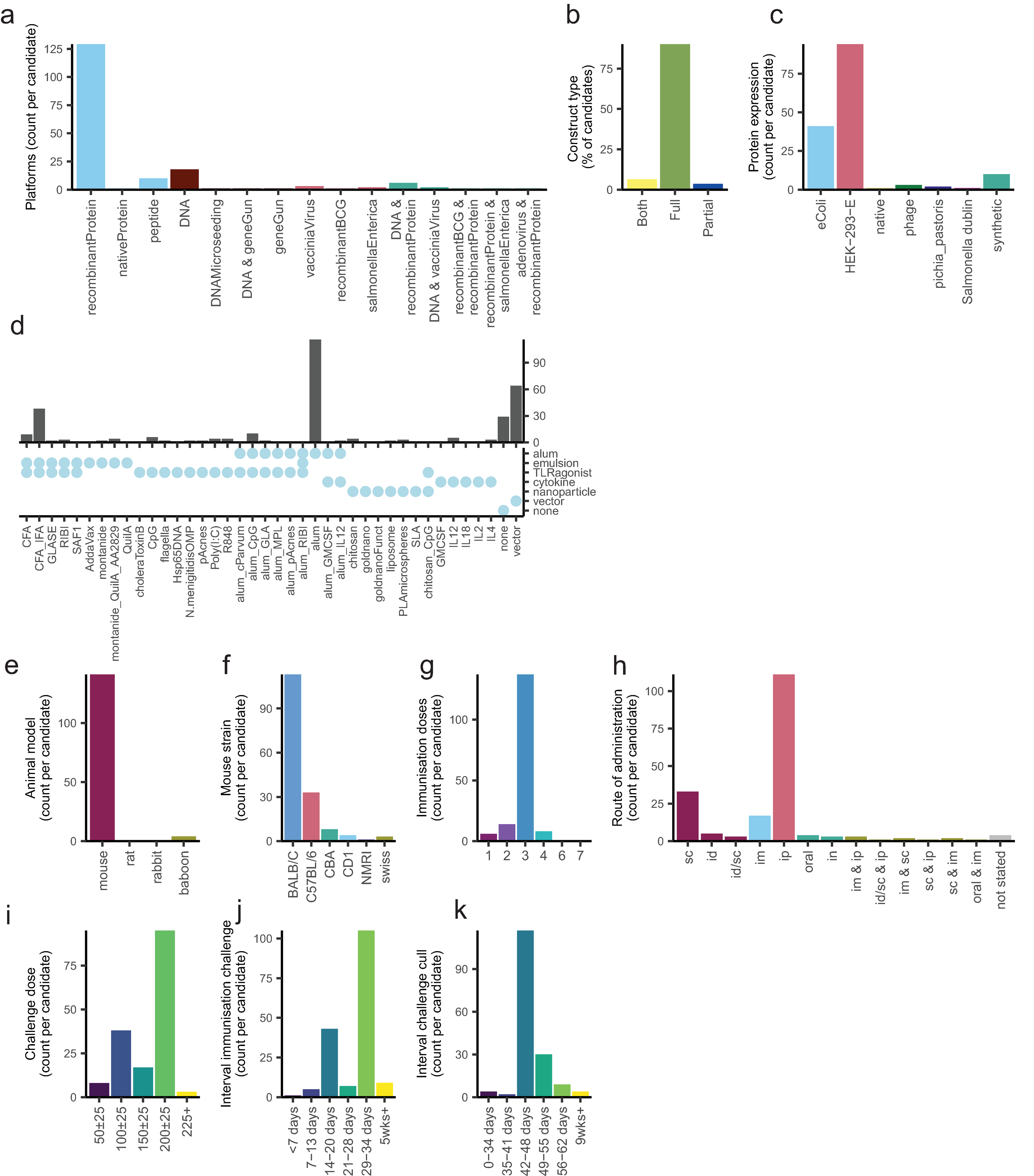

Supplement: S2 Fig — a) Platforms used for each schistosome vaccine antigen. b) Proportion of candidates tested as full constructs, partial constructs or both. c) Barplot showing recombinant protein system used for each protein based tested antigen. d) Bar and dotplot showing adjuvants used for each tested antigen, with adjuvant characteristics indicated on the dotplot below. e) Barplot showing animal model used for each tested antigen. f) Barplot showing mouse strain used for each antigen tested in a mouse model. g) Barplot showing the number of immunisation doses used for each tested antigen. h) Barplot showing administration route for each vaccine antigen. i) Barplot showing cercarial challenge dose for each vaccine antigen. j) Barplot showing gap between final immunisation and challenge for each vaccine antigen. k) Barplot showing gap between cercariae challenge and cull (measurement of worm/egg counts) for each vaccine antigen. (TIF) [file pntd.0012956.s003.tif]
