# Supplementary material for: Pre-clinical studies of Schistosoma mansoni vaccines: A scoping review
Source: PLoS Negl Trop Dis. 2025 Jun 2;19(6):e0012956. doi: 10.1371/journal.pntd.0012956 (PMC12158002; doi:10.1371/journal.pntd.0012956)
Supplement: Attachment — Submitted filename: Response to Reviewers.docx [file pntd.0012956.s006.docx]

Dear Editors and Reviewers,

Thank you for giving us the opportunity to submit a revised draft of our manuscript for publication in PLOS NTDs. We are very grateful for the time and effort you and the reviewers have put into assessing the manuscript and providing insightful feedback. Changes to the manuscript as a result of reviewer comments are shown as tracked changes in the marked version “Revised Manuscript with Track Changes”. Please see below our point-by-point answers (*in* *green*) to the reviewers concerns. Line numbers are accurate in the unmarked manuscript (“Manuscript”). We believe the manuscript has been substantially improved by the comments.

Yours sincerely,

Emma L. Houlder

## Reviewers' comments:

### Methods:

Reviewer #1: The review methods and statistics are appropriate

Reviewer #2: This paper is comprehensive and provides significant added value to the literature. Many reviews of Schistosoma vaccines exist, but none systematically compare different vaccines and vaccine parameters. This work will be invaluable for investigators designing pre-clinical vaccine trials and selecting antigens to push into clinical trials.

The review's objectives are articulated and provide a comprehensive overview of the current landscape in schistosomiasis vaccine development.

- *Thank you both very much for the positive comments.*

### Results:

Reviewer #1: The analysis resulted in excellent recommendations

Reviewer #2: The authors have clearly outlined a systematic and organized approach to reviewing preclinical data, and they remain consistent with that framework throughout the manuscript.

The table and figures are easy to interpret and contribute meaningfully to understanding the manuscript.

It would be helpful to note which vaccines have been tested with adjuvants/platforms that are approved for human use (or have a corollary like AddaVax/MF59). It is very relevant to the translatability of a vaccine candidate if its maximum efficacy is achieved with a system usable in humans versus one that is not.

Another variable that could be considered is whether any candidates have been tested independently by multiple research groups.

- *Thank you both very much for the positive comments. We completely agree that using a human-applicable adjuvant system is relevant to the translatability of a vaccine. We have now used bold to highlight human-applicable adjuvants in Figure 3g. In addition we have altered line 380 of the discussion to explicitly state that translatability of studies will increase if human-applicable adjuvants are used. Independent testing of candidates by different research groups is an interesting variable, and this has now been added to the summarising table (Table 1), discussed in line 182.*

### Conclusions:

Reviewer #1: The conclusions are relevant as the present the state of the field

Reviewer #2: They accurately synthesize the findings and highlight key trends in schistosomiasis vaccine research, including promising candidates and recurring challenges. The authors strike a good balance between optimism and realism, reflecting the field's current state.

- *Thank you both very much for the positive comments.*

### Editorial and Data Presentation Modifications?

Reviewer #1: Line 78; worm and/or egg counts. Recommend that both worm burden reduction and egg count be recommended. This reviewer realizes that egg count is a function of worm burden.

- *Worm or egg counts has been changed to worm and/or egg counts on line 78. The discussion section (line 323) recommends that both worm and egg counts are taken.*

Reviewer #2: Edits/minor changes

• Line 38: italicize Schistosoma *- changed*

• Line 43: comma after “design” *- changed*

• Sometimes there is a space between the end of the sentence and the parenthetical citation, sometimes there is not. *– Thank you for spotting this. We have now made this consistent.*

• Line 66: could be more clear to say “protection against schistosome infection” *- changed*

• Line 194: comma after “systems” *- changed*

• Line 195: comma after “efficacious” *– changed*

• Line 199-201: comma after “this” and/or could be reworded to be easier to follow *- this has now been re-phrased to be easier to follow.*

• Figure 4a: hamsters are mentioned in the text but not on the graph *– hamsters now changed to baboons to match the graph, thank you for pointing out this error.*

• Figures in general: it is not entirely clear to me what “count per candidate” refers to – is this the number of tests, the number of papers, etc.?

- *Thank you for your feedback, this is important to clarify. For “count per candidate” each test of a candidate is counted once per group (e.g. platform). Some candidates are tested repeatedly with the same parameters (for instance Sm-14 has been tested 31 times in a protein-based platform). The count per candidate metric was chosen to reduce the over-representation of these tests on the overview graphs, and thereby provide a hopefully more balanced overview. I hope this now makes sense. I have added the following to figure legends 3 and 4: “When count per candidate is plotted, each candidate is counted once per group (e.g. platform) to avoid over-representing frequently tested candidates and provide a balanced overview.”*

• Line 239: comma after “route” *- changed*

• Line 281: delete extra parenthesis *- changed*

• Line 294: comma after “parasite” *- changed*

• Line 413: add “are” between “post-vaccination” and “crucial” *- changed*

### Summary and General Comments:

Reviewer #1: The goal of this review is to present an overview of pre-clinical prophylactic vaccine studies targeting S. mansoni in the last 30 years (1994-2024). The article summarizes what is known about the antigens tested, their overall efficacy, the adjuvant and platform used, and the timing of immunization and challenge. Importantly the authors evaluate how the choice of these parameters may influence the efficacy reached in preclinical trials and discuss how these parameters translate to clinical vaccination trials.

Of the 141 vaccines tested that met the inclusion criteria only 10 achieved an efficacy of 60% and of the 10 only 2 reached an efficacy of >90% in pre-clinical studies. The outcome of the review of these vaccine preclinical trials is that to compare trials, researchers must consider employing consistent and optimized vaccine formulation and study design parameters.

One of the strengths of the review is that the authors have summarized in Tables the formulations and study designs used, and highlighted how certain parameters affect efficacy and with that information have made recommendations to researchers on how to perform vaccine tests in the future. This information will be useful for established labs to rethink their approach but also for new investigators.

A weakness is that the study focuses only on S. mansoni and the mouse model. Never-the- less, it is useful to point out the state of the vaccine field.

The outcome of the study is that development of a vaccine for schistosomiasis is not likely in the near future unless laboratories decide on consistent and comparable approaches. However, innovation is strongly encouraged.

Reviewer #2: This well-written and timely review offers a comprehensive and insightful synthesis of current progress in developing schistosomiasis vaccines. The manuscript is well-structured and logically organized and reflects a thorough command of the existing literature. It effectively highlights key advances in preclinical studies and provides a thoughtful analysis of the challenges.

- *Thank you both very much for the positive comments, I am glad that you believe this is a useful addition to the literature.*
